# Supplementary material for: Syngeneic AAV Pseudo-particles Potentiate Gene Transduction of AAV Vectors
Source: Mol Ther Methods Clin Dev. 2016 Dec 24;4:149–58. doi: 10.1016/j.omtm.2016.12.004 (PMC5363323; doi:10.1016/j.omtm.2016.12.004)
Supplement: Document S1. Figure S1 and Tables S1 and S2 [file mmc1.pdf]

OMTM, Volume *4*

Supplemental Information

Syngeneic AAV Pseudo-particles Potentiate Gene Transduction of AAV Vectors

Qizhao Wang, Biao Dong, Katie A. Pokiniewski, Jenni Firrman, Zhongren Wu, Mario P.S. Chin, Xiongwen Chen, LinShu Liu, Ruian Xu, Yong Diao, and Weidong Xiao

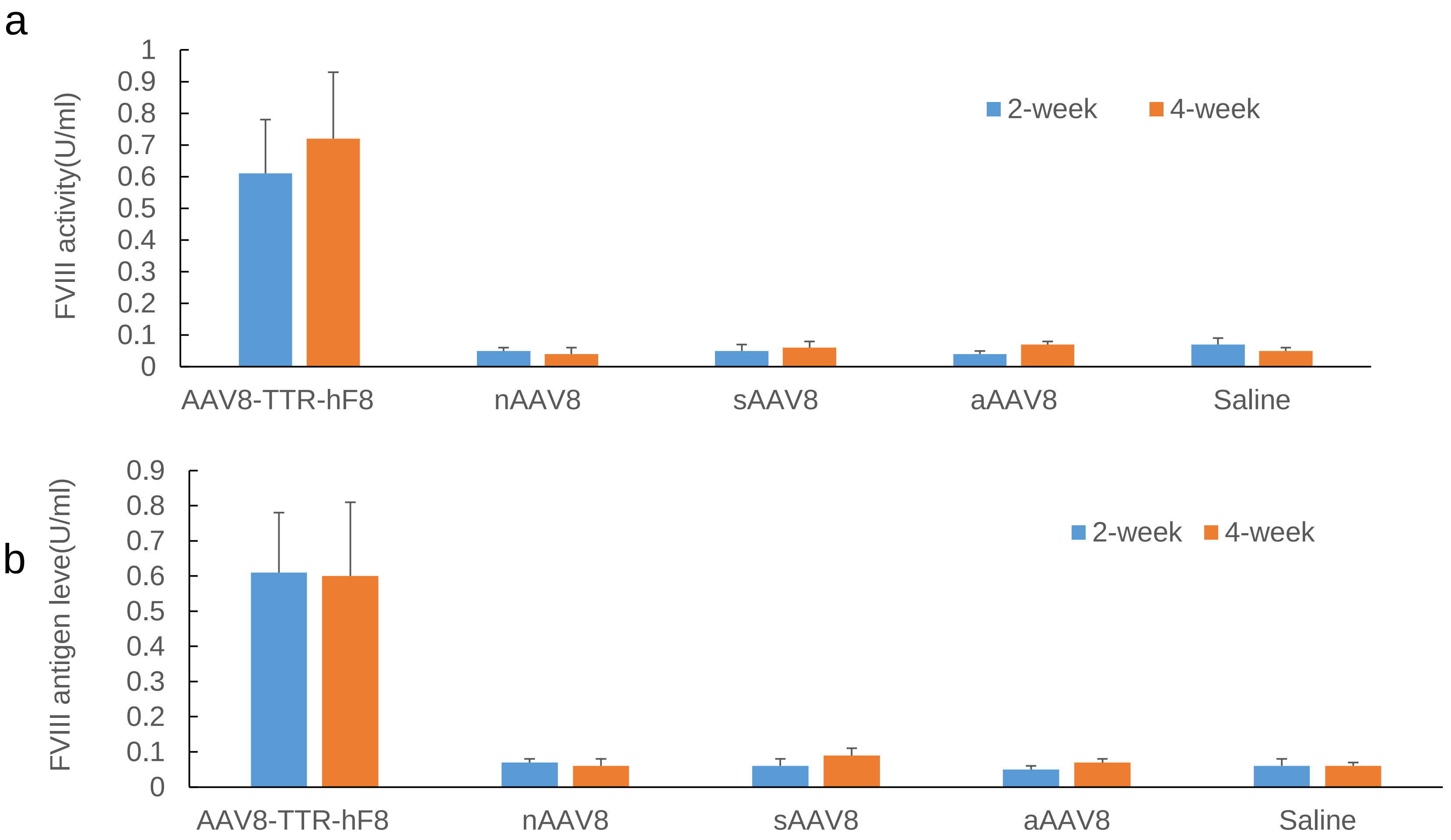

Figure S1. sAAV8 alone failed to express transgene *in vivo*. Blab/C mice were i.v. injected with AAV8-TTR-hF8 vectors (2×10^{11} vg/mouse) or 9X of AAV8 pseudo-vectors (nAAV8, sAAV8 and aAAV8). Transgene expression was detected by aPTT (a) and Elisa (b) at different points. nAAV, null AAV pseudo-vector; sAAV, syngeneic AAV pseudo-vector; aAAV, allogeneic AAV pseudo-vector.

Table S1. Probes for hybridization of AAV-TTR-hF8 and AAV-hHC genomes

Probe name	Targeting elements	Probe sequences	Location at AAV-hHC	Location at AAV-TTR-hF8
A	ITR	cgagcgagcgcgcagagagggagtggccaa-DIG	96-125; 3643-3672	96-125; 5051-5080
B	ApoE	acgcgtctgcaggctcagaggcacacagga-DIG	146-175	/
C	hHC	tccttgatgcaggatagggatgctgcatct-DIG	1593-1622	1201-1230
D	PolyA	gagatctgtgtgttggttttttgtgtgcgg-DIG	3494-3523	4902-4931
E	TTR	tgtctgtctgcacatttcgtagagcgagtg-DIG	/	151-180
F	hLC	agaaactgcagggctccctgcaatatccag-DIG		3565-3494

Table S2. qPCR primers for titration of AAV vectors

Primer pairs	Targeted genome	Forward primer	Reverse primer	PCR product (bp)	Location at AAV-hHC	Location at AAV-TTR-hF8
1	AAV-hHC; AAV-TTR-hF8	CGGCCTCAGTGAGCGA	GGAACCCCTAGTGATGGAGTT	62	83-144	83-144
2	AAV-hHC	AGTGGCCAACTCCATCACTA	GAGCCCAGAAACTCCTGTGT	71	117-188	
3	AAV-hHC	GCCTCTGAAGTCCACACTGA	AGGGCTGTGTGTTTGCTGT	92	243-344	
4	AAV-hHC	ACCTCCAACATCCACTCGAC	GCGTCGACCACACTACCTAA	86	393-478	
5	AAV-hHC; AAV-TTR-hF8	TCCACCTGCTTCTTTCTGTG	TCCACTGCACCCAGGTAGTA	74	909-982	517-590
6	AAV-hHC; AAV-TTR-hF8	CTGAAATGGATGTGGTCAGG	AGTCCCAGTCCTCCTCTTCA	126	2008-2133	1616-1741
7	AAV-hHC; AAV-TTR-hF8	AGTTCCAAGCCTCCAACATC	GGAAGTCAGTCTGTGCTCCA	126	2770-2895	2378-2503
8	AAV-hHC	CAGAATCCACCAGTCTTGA	GTCAATTTCCTCTTGATCTGAC	78	3180-3257	
9	AAV-hHC; AAV-TTR-hF8	CCTCTACTGACTCGAGAATAAA	GCCAACTCCATCACTAGG	95	3458-3552	4866-4690
10	AAV-TTR-hF8	AGTGGCCAACTCCATCACTA	ACCTTGCCTAGGGAGATTAG	94		117-210
11	AAV-TTR-hF8	CAGAATCAGCAGGTTTGGAG	GAACCACACACGGCACTTAC	144		261-404
12	AAV-TTR-hF8	CCAGATGGAAGATCCCACTT	GCTGAGCAGATACCATCGAA	124		3591-3714
13	AAV-TTR-hF8	GGCCATCAGTGGACTCTCTT	AATTCGAAGGTAGCGAGTCAG	123		4678-4800
qEGFP	AAV-CB-EGFP	TGACCCTGAAGTTCATCTGC	GAAGTCGTGCTGCTTCATGT	125	/	/
qCluc	AAV-CB-Cluc	TAAGACTTGCGGTATTTGCG	GCAGAGTCGTTCAGCTTCAG	135	/	/
