# Supplementary material for: Syngeneic AAV Pseudo-particles Potentiate Gene Transduction of AAV Vectors
Source: Mol Ther Methods Clin Dev. 2016 Dec 24;4:149–58. doi: 10.1016/j.omtm.2016.12.004 (PMC5363323; doi:10.1016/j.omtm.2016.12.004)
Supplement: Document S2. Article plus Supplemental Information [file mmc2.pdf]

Molecular Therapy
Methods & Clinical Development
Original Article

Syngeneic AAV Pseudo-particles Potentiate Gene Transduction of AAV Vectors

Qizhao Wang,[1,2] Biao Dong,[2] Katie A. Pokiniewski,[3] Jenni Firrman,[3,5] Zhongren Wu,[2] Mario P.S. Chin,[1] Xiongwen Chen,[4] LinShu Liu,[5] Ruian Xu,[1] Yong Diao,[1] and Weidong Xiao[1,2,3,4,5]

[1]School of Biomedical Sciences, Huaqiao University, Quanzhou, Fujian 362021, China; [2]Sol Sherry Thrombosis Research Center, Temple University, Philadelphia, PA 19122, USA; [3]Department of Microbiology and Immunology, Temple University, Philadelphia, PA 19122, USA; [4]Cardiovascular Research Center, Temple University, Philadelphia, PA 19122, USA; [5]United States Department of Agriculture, ARS, ERRC, Wyndmoor, PA 19038, USA

Adeno-associated virus (AAV) vectors have emerged as a safe and efficient gene therapy platform. One complication is that a significant amount of empty particles have always been generated as impurities during AAV vector production. However, the effects of such particles on AAV vector performance remain unclear. Here we systemically evaluated the biological properties of three types of "empty" AAV particles: syngeneic pseudo-vectors with partial AAV genomes derived from DNA of the corresponding full particles, allogeneic pseudo-vectors with partial genomes different from the corresponding full particles, and null pseudo-vectors with no DNA inside the capsids. The syngeneic particles in excess increased the corresponding full AAV vector transgene expression both in vivo and in vitro. However, such effects were not observed with null or allogeneic particles. The observed differences among these pseudo-AAV particles may be ascribed to the syngeneic pseudo-vector DNA facilitating the complementary DNA synthesis of the corresponding full AAV particles. Our study suggests that the DNA content in the pseudo-vectors plays a key role in dictating their effects on AAV transduction. The effects of residual "empty" particles should be adequately assessed when comparing AAV vector performance. The syngeneic AAV pseudo-vectors may be used to enhance the efficacy of gene therapy.

INTRODUCTION

Adeno-associated virus (AAV) is a non-pathogenic parvovirus with a 4.7-kb single-stranded DNA (ssDNA) viral genome that encodes two large open reading frames (ORFs) flanked by two inverted terminal repeats (ITRs). Exciting progress has been made using AAVs as gene delivery vectors for genetic diseases such as congenital blindness[1–3] and hemophilia.[4–6]

One major concern regarding AAV vectors used clinically is the presence of excessive empty capsids derived from typical AAV vector production processes.[7–10] Such empty AAV particles were also present in wild-type AAV (WTAAV) grown in the presence of adenovirus, which are generally referred as defective interfering AAVs because they can inhibit the replication of the WTAAV and adenovirus.[11,12] Although these particles appear "empty" under the electron microscope, they may contain DNA that is shorter than the full viral genome,[13,14] so they are called pseudo-AAV particles here. The recombinant AAV vectors usually generate even more pseudo-vector particles than WTAAV, especially those with oversized AAV genomes. The pseudo-vector-to-full vector ratios vary widely from 3:1 to 30:1, depending on the production and purification procedure employed.[8] It has been reported that, in some clinical-grade AAV vectors, the pseudo-vector content was as high as over 90%.[4]

It is speculated that the presentation of pseudo-vectors in clinic-grade preparations can substantially increase the amount of AAV capsid proteins and potentially lead to unwanted immunological consequences. One example of such side effects was observed during a clinical trial for the treatment of hemophilia with an AAV8 vector that contained a 10-fold excess of empty capsids over the full vector.[4] Recently, Gao et al. demonstrated that pseudo-vectors suppressed transgene expression and contributed to hepatic transaminase elevation in mice.[15] Moreover, the pseudo-vectors cogenerated and separated from the bona fide AAV8 vectors, resulting in higher elevations of liver enzyme than the vector alone or mixed with a completely empty vector. Hence, it is commonly accepted that removal of empty capsids from vector preparations should be beneficial, especially for clinical-grade AAV vectors.

However, it remains unclear whether pseudo-vectors are beneficial or detrimental to the clinical outcome. A recent report by Mingozzi et al. demonstrated that AAV8 pseudo-vectors can actually enhance gene transfer by overcoming the pre-existing humoral immunity to AAV8.[16] Even in situations without pre-immunization with intravenous immunoglobulin (IVIG), a 10-fold excess of pseudo-vectors would not significantly decrease the full AAV vector transduction. In nonhuman primates, the formulation of full AAV8-human FIX

Received 28 November 2016; accepted 16 December 2016;
http://dx.doi.org/10.1016/j.omtm.2016.12.004.

Correspondence: Yong Diao, School of Biomedical Sciences, Huaqiao University, Quanzhou, Fujian 362021, China.
E-mail: diaoyong@hqu.edu.cn
Correspondence: Weidong Xiao, Sol Sherry Thrombosis Research Center, Temple University, Philadelphia 19122, PA, USA.
E-mail: wxiao@temple.edu

This is an open access article under the CC BY license (http://creativecommons.org/licenses/by/4.0/).

(hFIX) vectors with 9× AAV8 pseudo-vectors (1.8 × 10^{13} particles/kg) was impervious. In a clinical study using AAV2 vectors expressing human factor IX (hFIX) to treat hemophilia B, wherein the pseudo-vectors were carefully removed, a capsid-specific T cell response was still observed.[17] Wu et al. recently showed that AAV8 pseudo-vectors were relatively poor stimulators of CD8+ T cells; the extent and duration of the CD8+ T cell response was influenced by the AAV vectors' genome.[18] Therefore, the effect of the presence of pseudo-vectors on clinical outcome remains an unsettled issue.

Several key questions remain unanswered, such as the mechanism of whether and how partially empty particles contribute to AAV vector transduction, which precise composition or structure is the cause, and whether we can formulate the pseudo-vectors into the final AAV vectors. Based on the DNA components in the AAV capsids, we classified the AAV pseudo-vectors as syngeneic AAV pseudo-vector (sAAV), allogeneic AAV pseudo-vector (aAAV), and null AAV pseudo-vector (nAAV). In the current study, we systemically analyzed the physical properties and effects of AAV pseudo-vectors on AAV vector transduction performance.

RESULTS

Syngeneic AAV8 Pseudo-vectors Enhance Transduction of the Corresponding Full Vectors In Vivo

To investigate whether pseudo-vector AAV particles reduce transduction efficiency in vivo, three different pseudo-vectors (sAAV8, nAAV8, and aAAV8) were generated and intravenously injected into a BALB/c hemophilia A (HA) mouse model together with full AAV8 vectors. The purified full AAV8 vector was AAV8-TTR-hF8 (5.1 kb)[19] (Figure 4A). sAAV8 was a by-product purified during the AAV8-TTR-hF8 production process, which carried partial genomes of AAV-TTR-hF8 (Figures 3 and 4). nAAV8 was produced from HEK293 cells transfected with the AAV8 packaging and adenoviral helper plasmids only, which do not carry any genomic DNA of the AAV vector. aAAV8 was a by-product of AAV8-hHC production, whose genome (Figure 4A) carries the human heavy chain (hHC) of the hF8 gene driven by the AAT promoter in combination with an ApoE enhancer. aAAV8 was named allogeneic AAV pseudo-vector in reference to sAAV8 because it was derived from AAV8-hHC instead of AAV8-TTR-hF8. The full AAV8-TTR-hF8 vectors (2 × 10^{11} vector genomes [vg]/mouse) were formulated in 1×, 3×, or 9× excess of nAAV8, sAAV8, or aAAV8 pseudo-vectors before co-injection.

As shown in Figure 1A, formulation of the full AAV8-TTR-hF8 vector with 1×, 3×, or 9× excess sAAV8 enhanced hF8 coagulation activity in a dose-dependent manner. This was confirmed by measuring the hF8 antigen level in mouse plasma using a HC-specific ELISA (Figure 1B). Formulation with 9× excess sAAV8 increased transgene expression by 150% and 62.2% 4 weeks post injection, as measured by both activated partial thromboplastin time (aPTT) assay and ELISA, respectively. 9× sAAV8 alone failed to produce detectable hF8, as measured either by aPTT assay or ELISA (Figure S1), suggesting that the increased hF8 expression level did not arise from sAAV8 itself. On the contrary, formulations with nAAV8 gave rise to moderate levels of coagulation activities of hF8 in plasma in HA animals, which is comparable with mice injected with full AAV8-TTR-hF8 vector alone (control). Similar to nAAV8, addition of 1× and 3× aAAV8 did not have any effect on AAV8-TTR-hF8 transduction in vivo. Although formulation of 9× excess of aAAV8 increased transduction by 50%, as measured using an aPTT assay at 4 weeks, no significant difference was observed when measured by ELISA. Those results suggest that the actual DNA composition of pseudo-vectors might contribute to their varied effects on full AAV8 vector transduction in vivo.

The effect of AAV pseudo-vectors on the liver transduction of full AAV8 vectors was further evaluated in C57BL/6 mice. 9× various pseudo-vectors were mixed with full AAV8-TTR-hF8 vector (2 × 10^{11} vg) and injected into mice intravenously. The presence of the nAAV8 and aAAV8 pseudo-vectors had no effect on AAV8-TTR-hF8 vector transduction in vivo (Figures 1C and 1D), which was consistent with a previous report.[16] However, the formulations with 9× sAAV8 resulted in a 3- to 5-fold increase in coagulation activity and 2- to 3-fold increase in antigen level. Taken together, these results demonstrated that the presence of sAAV8 pseudo-vectors in the preparation of full AAV8 vector enhanced transgene expression in vivo. This result was confirmed in two different mouse strains.

Syngeneic AAV8 Pseudo-vectors Enhance the Transduction of Full AAV Vectors In Vitro

To understand the mechanism of the enhancing effects of the pseudo-vectors on AAV transduction, the effects of various pseudo-vectors were analyzed in in vitro-cultured cells. As shown in Figure 2, the sAAV8 particles derived from pAAV-CB-Cluc, in which the *Cluc* gene is driven by a CB promoter, was prepared. nAAV8 and aAAV8 were the same as mentioned in the above in vivo experiments. 9× pseudo-vectors (9 × 10^4 particles/cell) were mixed with 1× AAV8-CB-Cluc (1 × 10^4 vg/cell) and then co-infected in HeLa-S3 cells. As shown in Figure 2A, neither nAAV8 nor aAAV8 influenced the full AAV8 vector transduction in vitro. In contrast, sAAV8 increased Cluc expression by 34.7% (Figure 2A). To further characterize the effects of DNA content, 3.4-kb AAV8-CB-EGFP was co-infected with nAAV8, sAAV8, and aAAV8 (Figure 2B). 9× sAAV8, derived from AAV8-CB-EGFP, resulted in a statistically significant increase in EGFP expression in GM16095 cells, whereas nAAV8 and aAAV8 showed no effect on AAV8-CB-EGFP transduction. These studies suggested that only sAAV8 pseudo-vectors derived from the bona fide full vectors, not aAAV8 and nAAV8, had the potential to enhance the corresponding full particle transduction.

Biological Properties of AAV8 Pseudo-vectors

To elucidate the biological properties of the AAV pseudo-vectors, we first examined the morphology of those vectors using transmission electron microscopy (TEM). Most of the negatively stained

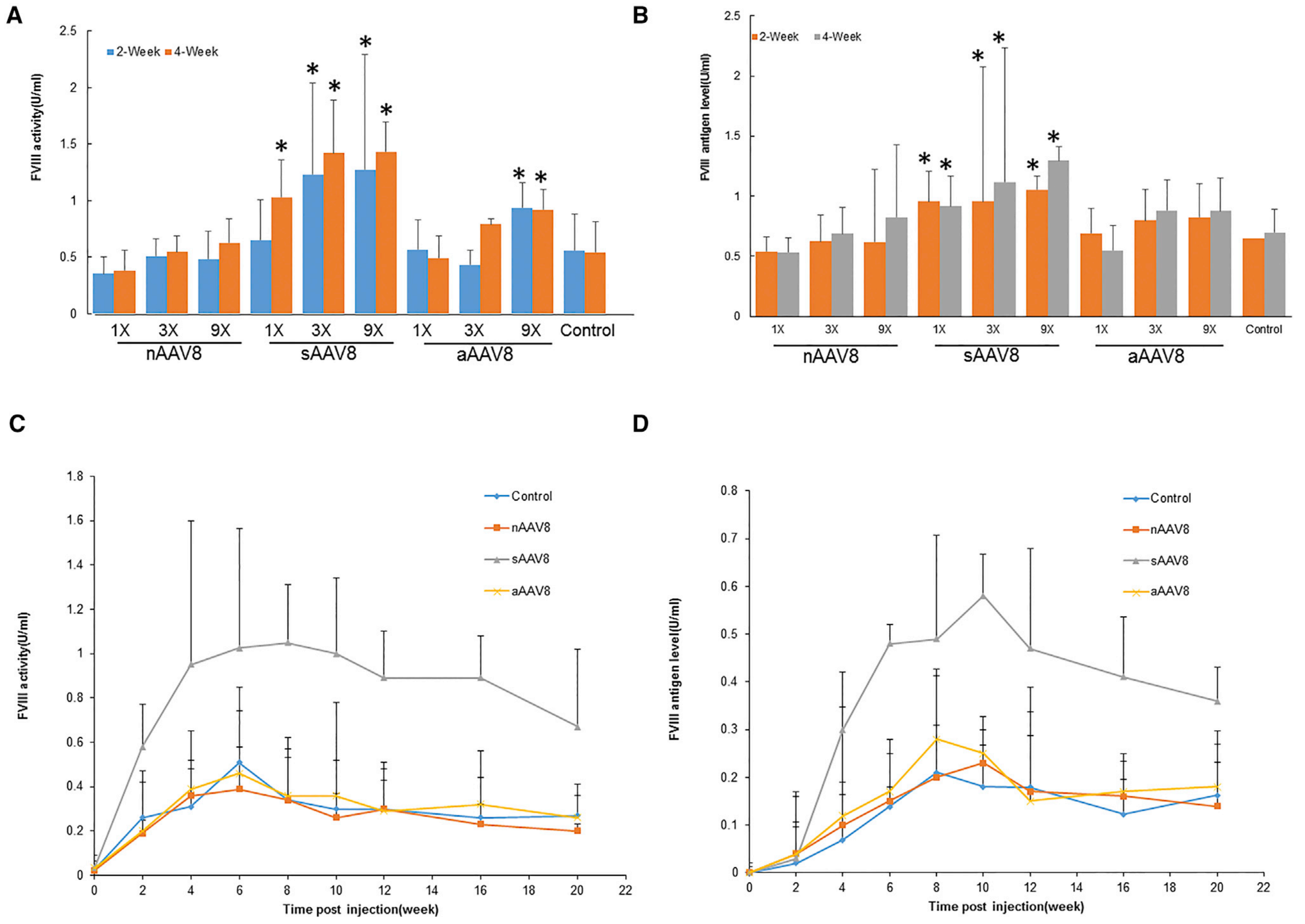

Figure 1. The Effects of Pseudo-vectors on Full AAV Transduction In Vivo

(A–D) Mice were i.v.-injected with AAV8-TTR-hF8 vectors (2×10^{11} vg/mouse) alone (control) or formulated with 1×, 3×, and 9× AAV8 pseudo-vectors (nAAV8, sAAV8, and aAAV8). Transgene expression was measured by aPTT (A and C) and ELISA (B and D) at different time points in BALB/c (A and B) and C57BL6/Svj129S (C and D) HA mice. n = 6, $^{*}p < 0.05$.

pseudo-vectors appeared to be empty because they displayed donut-like shapes of virions, and no significant difference was found among the three types of pseudo-vectors (Figure 3A).[15] We also did not observe self-assembly intermediates, which should be smaller than the regular particles.[20] Subsequently, we compared the capsid composition of those particles by silver staining. Besides the similarity in VP1, VP2, and VP3 capsid proteins between AAV8 pseudo-vectors and full vectors (Figure 3B), the additional VP1.5 band was also identical. The VP1.5 presumably arose from a G/C change at position 219 that introduced a novel CTG start codon for the polypetide.[10,21]

Although the morphology and capsid composition of the pseudo-vectors are indistinguishable from each other, we further analyzed the DNA content of these pseudo-vectors. As presented in Table 1, the DNA measured in our AAV8-TTR-hF8 vector preparation (full particles) is only equivalent to 63% of that expected from intact AAV particles with a complete 5.1-kb genome, suggesting that either it contained partial genomes, or pseudo-vectors were not completely removed. AAV8-hHC showed similar phenomena even though it is a regular-sized vector. In contrast to full AAV8 particles, lower DNA content was observed in both sAAV8 and aAAV8 particles (Table 1). sAAV8 contained 142 ± 51 ng DNA in 1.0E+13 particles, which is only about 0.51% of the theoretical value of the full AAV-TTR-hF8 vector genome, whereas aAAV8 contained 0.96% of the theoretical DNA of the AAV-hHC genome.

To assess the DNA content, the extracted DNA from sAAV8 and aAAV8 particles was analyzed by electrophoresis on a 1.5% agarose gel. As shown in Figure 3C, the extracted DNA was mostly less than 0.5 kb, with peaks at approximately 120 bp and 200 bp (Figure 3D). Under denaturing conditions, DNA fragments for both sAAV8 and aAAV8 particles were detected. Four major bands (~120 nt, ~340 nt, ~410 nt, and ~570 nt) and one minor band (~450 nt) were detected in aAAV8. In contrast, the DNA fragments in sAAV8 were distributed over a large range, resulting a more

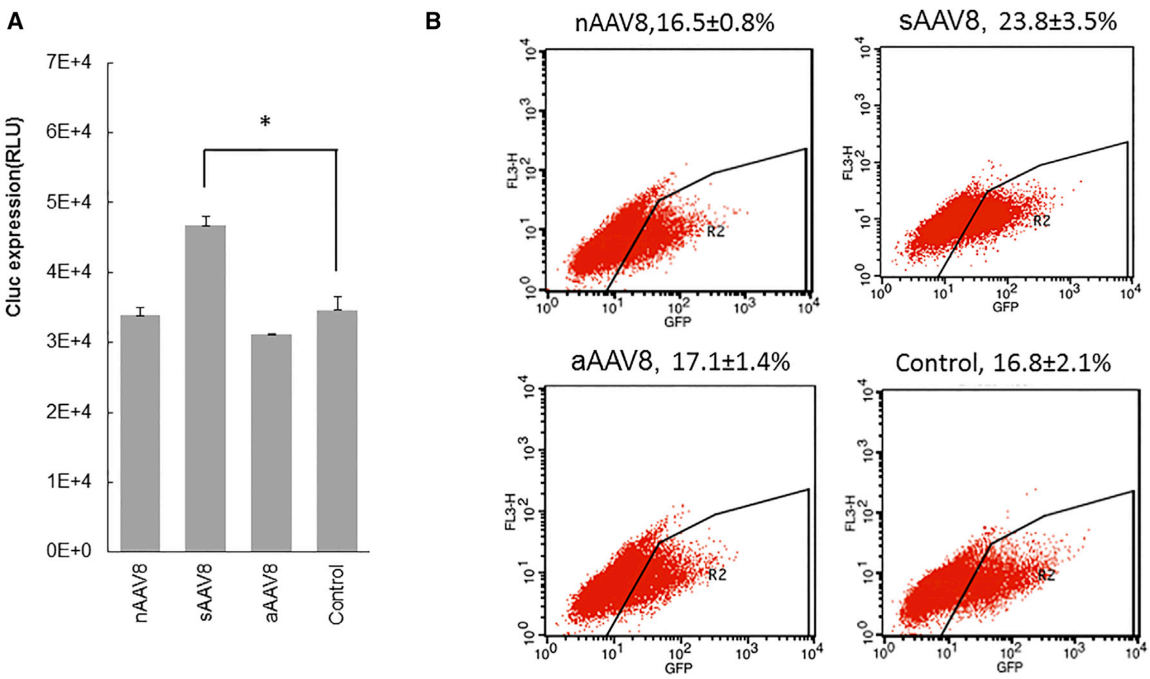

Figure 2. The Effects of Pseudo-vectors on Full AAV Transduction In Vitro

(A) HeLa cells were transduced with 1 × 10^4 vector genomes/cell of AAV8-CB-Cluc vector. 9× AAV pseudo-vectors were co-transduced with full AAV vectors. Luciferase activity was determined 36 hr post-transduction. (B) GM16095 cells were transduced with 1 × 10^4 vector genomes/cell of AAV8-CB-EGFP vector. 9× AAV pseudo-vectors with different resources were co-transduced. EGFP expression was quantified 48 hr post-transduction by flow cytometry. n = 6, *p < 0.05.

diffusing pattern. In summary, this study suggested that the AAV8 pseudo-vectors separated from the regular AAV vector purification procedures were not completely empty in that they carried varying DNA sequences.

The DNA Fragments in AAV Pseudo-vectors Contain AAV Inverted Terminal Repeats

To define the DNA composition in AAV pseudo-vectors, we assessed the relative abundance of representing DNA fragments throughout the entire recombinant AAV genome using oligonucleotides specific to a particular location (Figure 4A; Table S1). The DNAs isolated from sAAV8 and aAAV8 all hybridized strongly to the probe (A) specific to the ITR sequence (Figure 4B), whereas the probes (C and F) specific to the vector sequences in the middle region did not show a convincing hybridization signal at all. Our results demonstrated that most of the DNA fragments in sAAV8 and aAAV8 contained AAV ITRs, which is consistent with the theory that AAV encapsidation initiated from the 3′ ITR.

The DNA content of sAAV8 and aAAV8 was further assessed by qPCR. Thirteen pairs of primers spanning the whole vector sequences of AAV-TTR-hF8 and AAV-hHC were used to measure the relative abundance of specific DNA fragments in the AAV pseudo-vectors (Figure 4A; Table S2). A U-shaped plot was observed (Figures 4C and 4D), which demonstrated that AAV ITRs were the most abundant sequences in both sAAV8 and aAAV8. Few sequences corresponding to the midsection of the vector genome were detected, suggesting that the pseudo-vectors rarely contained sequences from the middle region.

sAAV8 Increased dsDNA Formation from Full AAV Particles

Because the AAV viral genome consists of ssDNA, which is transcriptionally inactive, it has to be converted to double-stranded DNA (dsDNA) to be transcribed. The transgene expression level is generally proportional to the amount of duplex vector genomes. Because sAAV pseudo-vectors contained a partial sequence derived from the vector genome, we hypothesized that these short DNA fragments may have annealed to the single-stranded DNA of the full AAV particles and, therefore, facilitate the synthesis of the complementary strand, resulting in the formation of dsDNA that can express transgenes. To test this hypothesis, we infected GM16095 cells with 9× AAV8 pseudo-vectors together with 1× full AAV8-CB-EGFP (1 × 10^4 vg/cell) vectors and examined the vector genome status by Southern blot (Figures 5A and 5B) and qPCR (Figure 5C). As expected, sAAV8 was able to increase the amount of dsDNA vector genome by 2∼3-fold, whereas aAAV8 and nAAV8 did not have similar effects. This increase in ds DNA vector genomes was consistent with the increase in EGFP-positive cells (Figure 2C). To confirm this observation in vivo, we analyzed the genomic DNA from the livers of mice that received an injection of AAV8-TTR-hF8 vector. As shown in Figure 5D, the

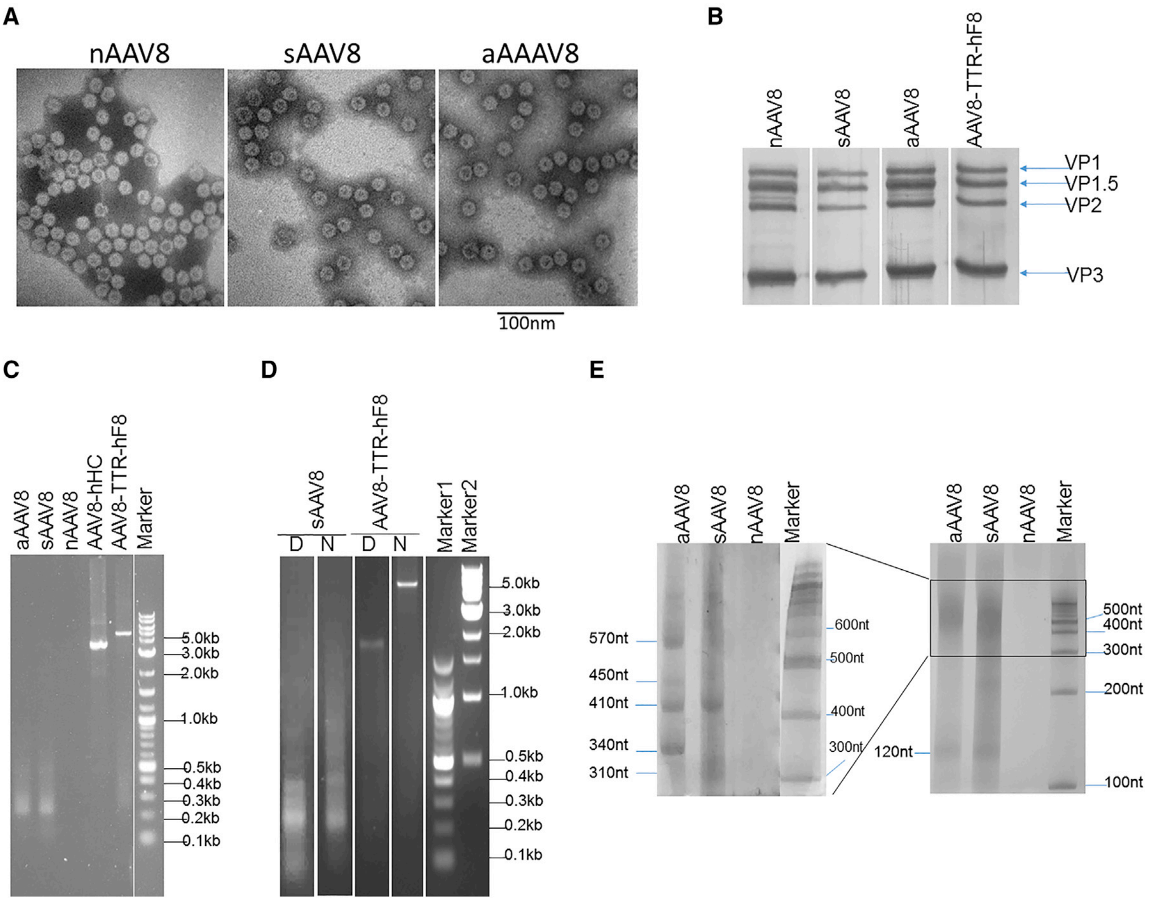

Figure 3. Characterization of AAV Pseudo-vectors

(A) Morphology of nAAV8, sAAV8, and aAAV8. Vector preparations were negatively stained with uranyl acetate and examined using TEM technology. The viral particles with the dimpled (dark) center are the pseudo-vectors. (B) Capsid composition of the pseudo-vectors. Approximately 1×10^{10} purified viral particles were denatured, electrophoresed on a 10% SDS-PAGE gel, and visualized using silver staining. VP1, VP2, VP3, and an additional VP1.5 band are identified. (C) Analysis of DNA genomes from empty AAV particles using agarose gel. (D) Size distribution of DNA genomes from AAV8 pseudo-vectors and full AAV8 genomes after denaturing treatments. (E) Analysis of specific size classes of DNA genomes from AAV8 pseudo-vectors using urea-denaturing SDS-PAGE.

hF8 copy number in the mouse receiving the coinjection of sAAV8 was 3-fold higher than in the control receiving the full vector particles only. Coinjection of nAAV8 or aAAV did not result in any significant increases in hF8 genomic copy number compared with the full vector alone.

DISCUSSION

AAV-based gene therapy clinical trials have yielded promising outcomes in treating genetic diseases. One major complication in research or clinical AAV vectors is that AAV vector-related impurities, empty particles closely resembling the normal vectors, are often

Table 1. DNA Amounts in AAV Pseudo-vectors and Full AAV Particles

			DNA Content (ng/1E+13 Particles)		
Vectors	Density (g/cm^3)	Genome Size (nt)	Theoretical Value	Experiment Value	Percent[a]
nAAV8	1.32–1.34	–	–	–	–
aAAV8	1.32–1.34	< 1,000	–	193 ± 46	0.96
sAAV8	1.32–1.34	< 1,000	–	142 ± 51	0.51
AAV8-TTR-hF8	1.40–1.47	5,080	27,847	17,543 ± 2,240	63.0
AAV8-hHC	1.38–1.44	3,672	20,129	17,588 ± 3,291	87.4

[a]Percent is the ratio of DNA amount in sAAV8 or nAAV8 to their theoretical value with 100% of AAV vectors containing the intact genome.

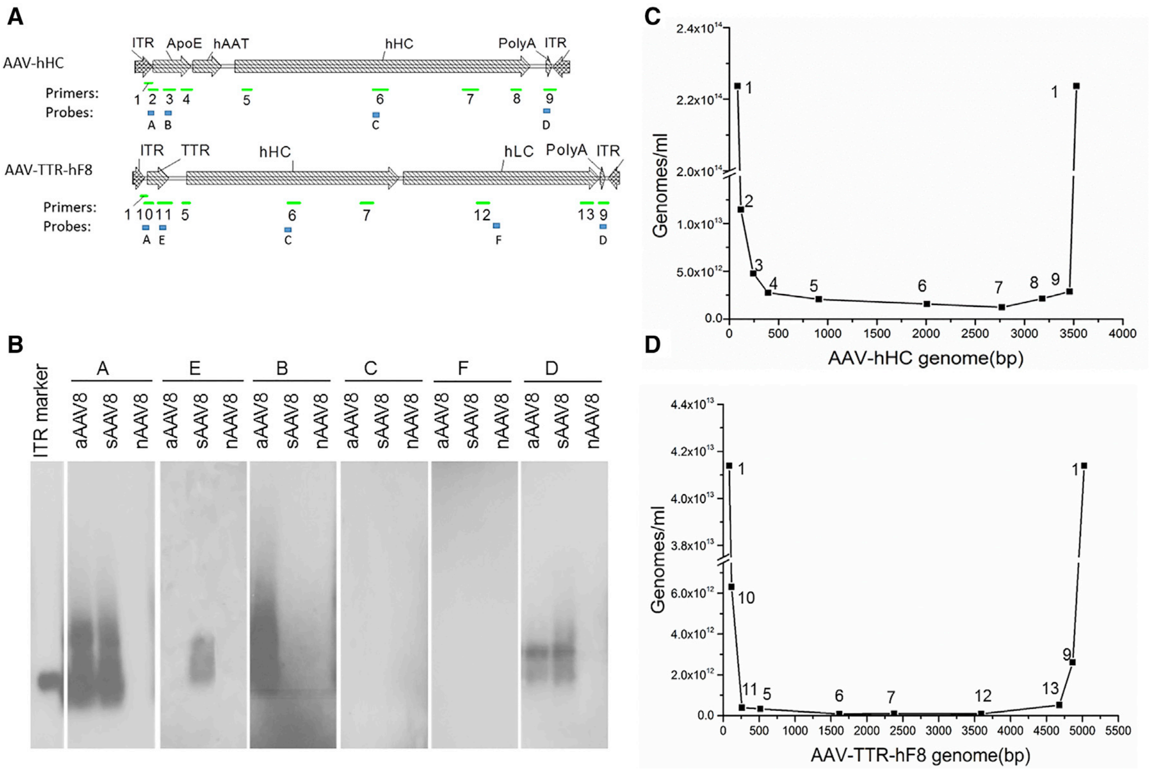

Figure 4. The AAV Pseudo-vectors Contain Inverted Terminal Sequences

(A) Primer and probe positions on AAV genomes. (B) Southern blot analysis of AAV pseudo-vector genome DNA with probes specific to different regions of the AAV genome. 1 ng DNA was loaded in each well. (C and D) qPCR analysis of AAV pseudo-vector genome DNA from AAV-hHC (C) and AAV-TTR-hF8 (D). The numbers annotated in the curves of (C) and (D) stand for the primer pairs illustrated in (A).

difficult to remove completely and, thus, are being injected along with the full vectors. The effects of such impurities (i.e., AAV pseudo-vectors, AAV defective interfering (DI-AAV) particles, or empty particles), have been largely derived from early studies of wild-type AAV, showing their abilities to inhibit AAV and adenovirus replication.[11,12] Hence, it appears logical to conclude that AAV pseudo-vectors would reduce the full AAV vector transduction by competing for receptor binding, internalization, and intra-cellular trafficking. However, recent studies showed that their functional effect on recombinant AAV vector transduction is controversial.[15]

In the present study, we provide evidence that the DNA content in the AAV pseudo-vector is the main factor that affects the transduction of the full AAV vector (Figures 1 and 2). Co-administration of sAAV8 carrying partial DNA derived from the corresponding full vector can enhance the efficacy of gene expression in a dose-dependent manner. This is in agreement with the observation from the recent AAV8-FIX trial for hemophilia B, in which up to 10× empty capsids may have increased the therapeutic effects.[4,17] In contrast, co-administration of excess aAAV8 or nAAV8 did not have any significant effect on the transduction of full AAV particles.

In contrast to nAAV pseudo vectors without any nucleic acid in the capsid, DNAs in sAAV and aAAV are structurally similar (i.e., short ITR-containing DNA fragments; Figures 3 and 4), which probably arises from the fact that AAV encapsidation initiates from the 3′ ITR and aborted packaging prematurely.[22] These short AAV DNA fragments anneal naturally to the 5′ end of single-stranded AAV genomes, which will not lead to the increase in second-stranded DNA synthesis. However, after conversion of the 3′ ITR fragments of the syngeneic pseudo-vector to a complimentary sequence, it can anneal to the corresponding AAV full genome and, subsequently, serve as 3′ primer for second-strand DNA synthesis (Figure 6A). In contrast, DNA from the allogeneic pseudo-vector is unrelated to the full vector, which cannot anneal to the full vector genomes and did not have any effect on transduction (Figures 1 and 2). Alternatively, the 3′ ITR fragments of the syngeneic pseudo-vector can anneal to the full vector genome that has already undergone its second-strand DNA synthesis and further increase the copy numbers of vector genomes that can express therapeutic genes (Figure 6B).

One concern about AAV pseudo-vectors in clinical application is the potential complication of inducing innate or adaptive immune responses.[4,15,17,18,23,24] The presence of pseudo-vectors increased the amount of AAV capsid antigen. It is also possible that AAV

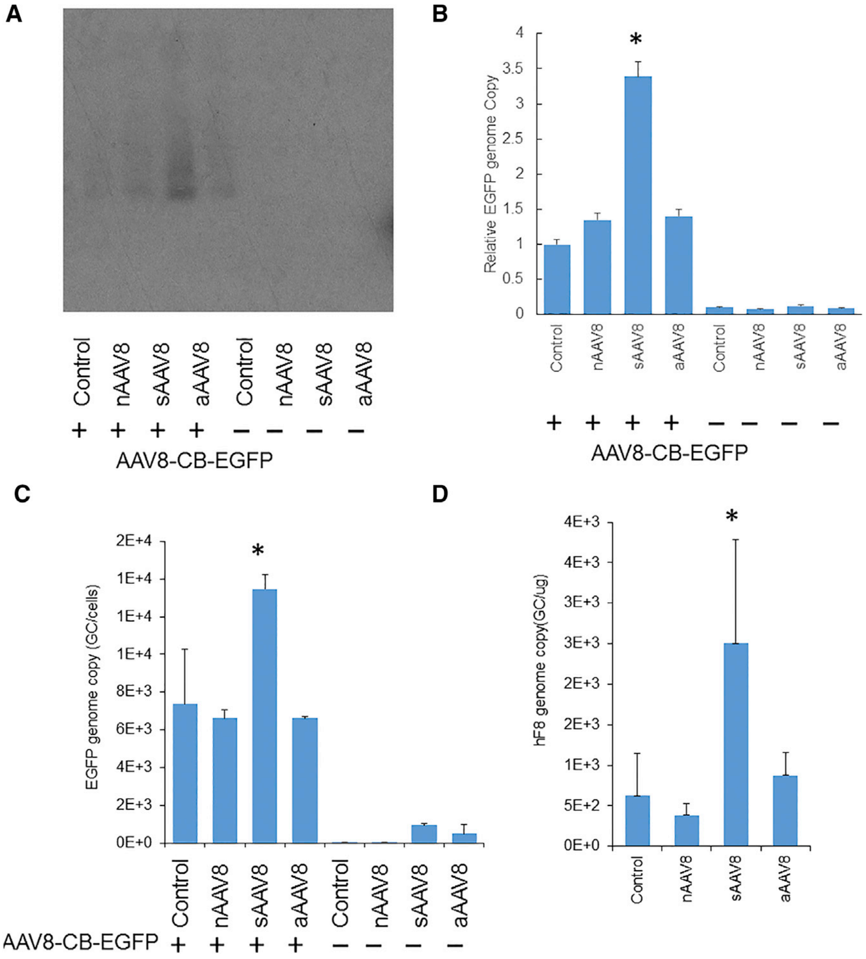

Figure 5. sAAV Pseudo-vectors Enhance Full AAV Vector Transduction by Increasing Vector Genomes

(A and B) Southern blot analysis of AAV DNA extracted from GM16095 cells that were infected with 9× nAAV8, aAAV8, or sAAV8 alone or together with 1× full AAV8-CB-EGFP (1 × 10^4 vg/cell) vectors. Hirt DNA was extracted 48 hr post AAV infection. Quantification of AAV genomes in (A) were obtained using ImageJ software, represented as fold change compared with cells only infected with 1× AAV8-CB-EGFP (B). (C) qPCR analysis of AAV genomes in GM16095 cells. (D) Vector gene copy number in mouse livers collected 18 weeks after AAV8-TTR-hF8 gene transfer at a vector dose of 2 × 10^{11}vg/mouse. Results are the average copy numbers of five mice.

pseudo-vectors may reduce transduction efficiency by competing for vector binding sites when vectors are administrated at a high dose when potential AAV receptors are saturated. AAV pseudo-vectors can also serve as efficient bait for AAV-specific antibodies that are prevalent in the human population.[16] Whether it is beneficial to include syngeneic pseudo-vectors in a clinical application is debatable despite the fact that it can increase therapeutic gene expression. However, because syngeneic pseudo-particle contamination is often present in the vectors for administration when using a common AAV purification protocol, their effects on preclinical studies are an important issue that should be carefully evaluated.

MATERIALS AND METHODS

Production and Purification of AAV Vectors and Pseudo-vectors

The pAAV-hHC, pAAV-CB-EGFP, pAAV-CB-Cluc, and pAAV-TTR-hF8 plasmids used for packaging the AAV vectors were described previously.[19,25] All AAV vectors were generated using the triple plasmid co-transfection method.[26,27] Briefly, pAAV-Rep-Cap (serotype 8), pAd helper, and the transgene plasmids were co-transfected into HEK293 cells and cultured in roller bottles at a ratio of 1:1:1. The vectors from transfected cells and medium were harvested 72 hr post transfection and purified by two rounds of CsCl gradient ultracentrifugation. sAAV8 was a by-product purified from its corresponding full AAV8 production process, and aAAV8 was a by-product generated from another full AAV8 containing a different genome. For example, while the empty AAV vectors generated in the AAV8-TTR-hF8 production process are syngenic to AAV8-TTR-hF8 itself (sAAV8), they are considered to be allogenic to the AAV8-hHC vector or other AAV8 vectors (aAAV8). By the same token, the empty AAV vectors purified from AAV8-hHC vector preparation are syngenic to AAV8-hHC(sAAV8), but allogenic to AAV8-TTR-hF8 or other AAV8 vectors (aAAV8). The nAAV8 pseudo-vectors were obtained by transfecting only pAAV-Rep-Cap and pAd helper plasmids into HEK293 cells. The AAV bands corresponding to the pseudo-vectors and full vectors in the CsCl gradient after ultracentrifugation were collected and extensively dialyzed against PBS containing 5% D-sorbitol.

Titration and Characterization of the AAV Capsid Using Silver Staining

The titers of AAV pseudo-vectors and the capsid composition of AAV vectors were determined by silver staining analysis. Briefly, AAV vectors (~1 × 10^{10} particles) were resolved by electrophoresis on a 10% SDS-PAGE gel, and standard silver staining was performed according to the manufacturer's procedures (Pierce Silver Stain Kit, Thermo Scientific). The titers of AAV pseudo-vectors were semi-quantified based on the titer of the full AAV, which was measured using qPCR analysis.

Analysis of AAV Pseudo-vector Genomes

AAV pseudo-vectors (~1 × 10^{13}) were treated with DNase I and proteinase K, and the DNA was extracted using a GeneJET gel purification kit. After extraction, 10 ng of each DNA sample, with or without denaturation treatment, was examined on a 1.5% agarose gel with

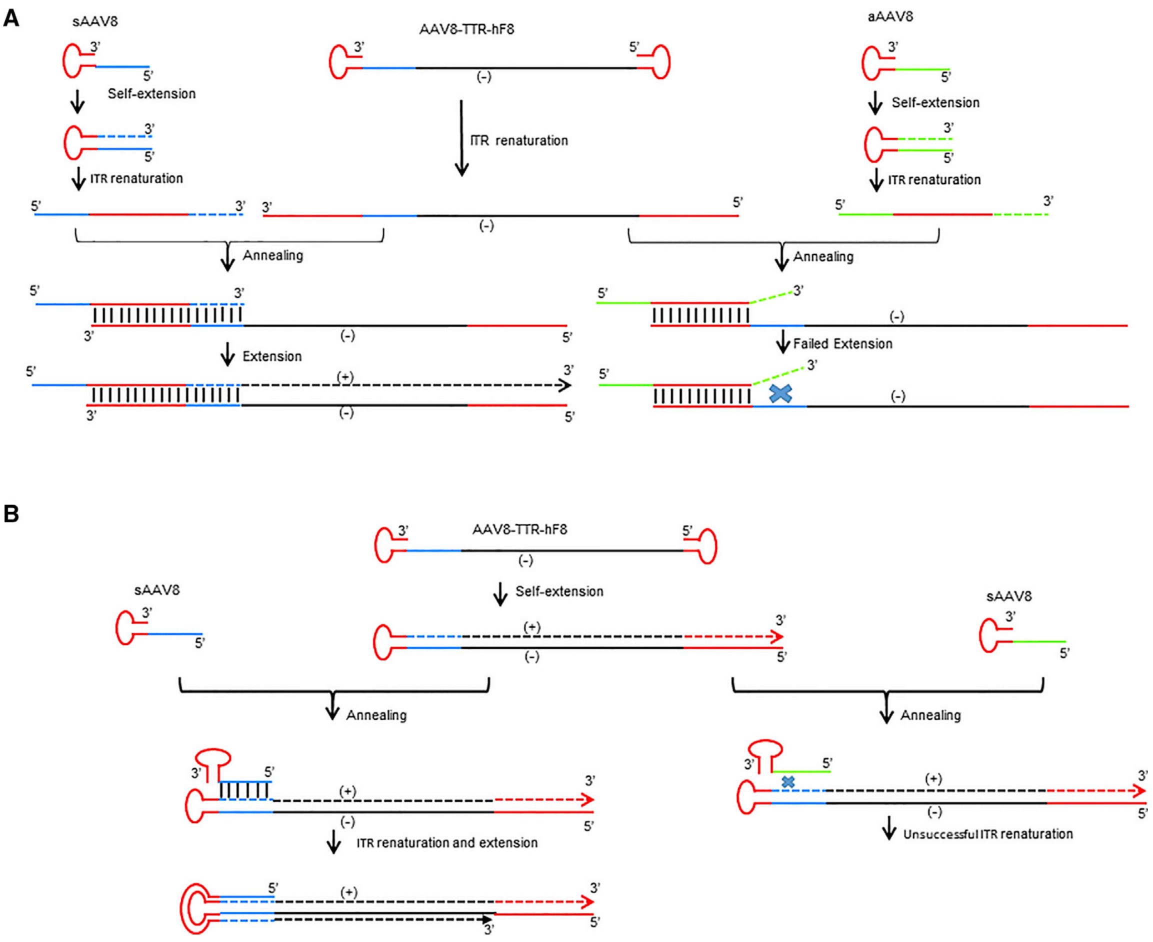

Figure 6. Proposed Models for AAV Pseudo-vector Enhancement AAV Transduction

(A) Self-extended sAAV genomes serve as primers for duplex vector DNA formation. DNA genomes from sAAV pseudo-vectors contain short ITR-associated sequences. (B) sAAV genomes serve as primers directly. They anneal with the new synthesized full AAV genomes and result in more dsDNA formation after extension.

SYBR Gold staining. The DNA from the AAV pseudo-vectors was analyzed by 6% SDS-PAGE gel containing 7 M urea and visualized with silver staining. The digoxigenin (DIG)-labeled oligonucleotides (Table S2) were utilized for DNA hybridization to detect the AAV genomes from the pseudo-vector.

qPCR Analysis of AAV Genomes from AAV Pseudo-vectors and Full Vectors

A 10-μL aliquot of full AAV vectors was treated with DNase I and proteinase K. After treatment, the DNA was diluted to different ratios and analyzed. The DNA content of the AAV pseudo-vectors was diluted and analyzed directly after extraction. To determine the copy number of the genome in the liver, total DNA was extracted from the liver by using a GeneJET genomic DNA purification kit (Thermo Scientific, K0721) according to the standard protocol. To evaluate the genome copy number in the AAV vector-transduced cells, the total DNA was extracted using Hirt extraction. The qPCR analysis was performed using Fast SYBR Green Master Mix, as described previously,[28] using the primers presented in Table S1. The genome copy number of the vector in each sample was determined by a standard curve obtained from the serial dilution of plasmids containing the corresponding AAV genomes.

Transmission Electron Microscopy

For TEM analysis, formvar was coated onto a microscope slide and floated onto a water bath. Cleaned 200-mesh grids were placed on this film and collected using parafilm. Five microliters of purified AAV (~1 × 10^{13} vg/ml) was placed on the grid and allowed to dry, and then 8 μl of 1% phospho-tungstic acid (Electron Microscopy Science) was applied and drawn off. After drying, the grids were observed using a Philips Transmission Electron Microscope CM 12 (Philips) with an accelerating voltage of 100 kV and imaged with a DVC detector controlled by AMT software.

rAAV Transduction In Vitro

The cell lines, HEK293, HeLa-S3, and GM16095, used in this study were grown in DMEM (Invitrogen) with 10% fetal bovine serum (FBS) (HyClone), penicillin (100 U/mL), and streptomycin

(100 μg/mL) at 37°C in a humidified environment supplied with 5% CO_2. For each transduction experiment, 50,000 viable cells were seeded in a 24-well plate 24 hr before transduction. AAV vectors, with or without pseudo-vectors, were added directly to each well. EGFP and Cluc expression was determined 48 hr post infection. EGFP expression was analyzed by flow cytometry, whereas Cluc expression from the medium was determined with the BioLux *Cypridina* luciferase assay kit and a POLARstar Omega bioluminescence plate reader (POLARstar Omega). All experiments were performed in triplicate, and the results are presented as the mean.

rAAV Transduction In Vivo

The in vivo transduction experiments were carried out with 6- to 8-week-old BALB/c and C57BL6/Svj129S male HA mice. The animals were housed in a specific pathogen-free environment and provided a normal diet. They were treated in accordance with NIH guidelines, and all protocols were approved by the Institutional Animal Care and Use Committee (IACUC) at Temple University. AAV8-TTR-hF8 (2×10^{11} vg/mouse) alone or formulated with 1×, 3×, and 9× AAV pseudo-vectors was injected into the mice via the tail vein as described previously.[28] Post vector administration, the plasma was harvested by retro-orbital bleeding at regular intervals as described. The biologically active hF8 in the plasma was determined by one-stage aPTT assay, and the protein level was estimated by ELISA using specific antibodies against hHC as described previously.[28] ReFacto (Wyeth) was used as the standard for the aPTT assay and ELISA.

Statistical Analyses

Two-tailed Student's t test and one-way ANOVA with Bonferroni multiple comparisons were performed. The differences were considered significant when $p < 0.05$. The analysis was conducted using SPSS 11.0.

SUPPLEMENTAL INFORMATION

Supplemental Information includes one figure and two tables and can be found with this article online at http://dx.doi.org/10.1016/j.omtm.2016.12.004.

AUTHOR CONTRIBUTIONS

Q.W. helped with study design, performed experiments and data analysis, and prepared figures and the manuscript. B.D., K.A.P., J.F., and Z.W. performed some of experiments. J.F. and L.L. performed TEM experiments. J.F., M.P.S.C., X.C., and R.X. helped with manuscript writing and editing. Y.D. and W.X. helped with study coordination and manuscript writing and editing. All authors reviewed and commented on the manuscript.

CONFLICTS OF INTEREST

The authors declare no conflicts of interest.

ACKNOWLEDGMENTS

This work is supported by NIH Grants HL080789, HL114152, and HL130871 and Natural National Science Foundation of China Grants 81271691, 81371669, and 81371672.

REFERENCES

1. Bennett, J., Ashtari, M., Wellman, J., Marshall, K.A., Cyckowski, L.L., Chung, D.C., McCague, S., Pierce, E.A., Chen, Y., Bennicelli, J.L., et al. (2012). AAV2 gene therapy readministration in three adults with congenital blindness. Sci. Transl. Med. *4*, 120ra15.
2. Cideciyan, A.V., Hauswirth, W.W., Aleman, T.S., Kaushal, S., Schwartz, S.B., Boye, S.L., Windsor, E.A., Conlon, T.J., Sumaroka, A., Pang, J.J., et al. (2009). Human RPE65 gene therapy for Leber congenital amaurosis: persistence of early visual improvements and safety at 1 year. Hum. Gene Ther. *20*, 999–1004.
3. Ku, C.A., and Pennesi, M.E. (2015). Retinal Gene Therapy: Current Progress and Future Prospects. Expert Rev. Ophthalmol. *10*, 281–299.
4. Nathwani, A.C., Tuddenham, E.G., Rangarajan, S., Rosales, C., McIntosh, J., Linch, D.C., Chowdary, P., Riddell, A., Pie, A.J., Harrington, C., et al. (2011). Adenovirus-associated virus vector-mediated gene transfer in hemophilia B. N. Engl. J. Med. *365*, 2357–2365.
5. Rogers, G.L., and Herzog, R.W. (2015). Gene therapy for hemophilia. Front. Biosci. (Landmark Ed.) *20*, 556–603.
6. High, K.A. (2014). Gene therapy for hemophilia: the clot thickens. Hum. Gene Ther. *25*, 915–922.
7. Wright, J.F. (2014). AAV empty capsids: for better or for worse? Mol. Ther. *22*, 1–2.
8. Qu, G., Bahr-Davidson, J., Prado, J., Tai, A., Cataniag, F., McDonnell, J., Zhou, J., Hauck, B., Luna, J., Sommer, J.M., et al. (2007). Separation of adeno-associated virus type 2 empty particles from genome containing vectors by anion-exchange column chromatography. J. Virol. Methods *140*, 183–192.
9. Urabe, M., Xin, K.Q., Obara, Y., Nakakura, T., Mizukami, H., Kume, A., Okuda, K., and Ozawa, K. (2006). Removal of empty capsids from type 1 adeno-associated virus vector stocks by anion-exchange chromatography potentiates transgene expression. Mol. Ther. *13*, 823–828.
10. Wang, Q., Firrman, J., Wu, Z., Pokiniewski, K.A., Valencia, C.A., Wang, H., Wei, H., Zhuang, Z., Liu, L., Wunder, S.L., et al. (2016). High density recombinant AAV particles are competent vectors for in vivo Transduction. Hum. Gene Ther. *27*, 971–981.
11. de la Maza, L.M., and Carter, B.J. (1981). Inhibition of adenovirus oncogenicity in hamsters by adeno-associated virus DNA. J. Natl. Cancer Inst. *67*, 1323–1326.
12. Carter, B.J., Laughlin, C.A., de la Maza, L.M., and Myers, M. (1979). Adeno-associated virus autointerference. Virology *92*, 449–462.
13. Senapathy, P., and Carter, B.J. (1984). Molecular cloning of adeno-associated virus variant genomes and generation of infectious virus by recombination in mammalian cells. J. Biol. Chem. *259*, 4661–4666.
14. de la Maza, L.M., and Carter, B.J. (1980). Molecular structure of adeno-associated virus variant DNA. J. Biol. Chem. *255*, 3194–3203.
15. Gao, K., Li, M., Zhong, L., Su, Q., Li, J., Li, S., He, R., Zhang, Y., Hendricks, G., Wang, J., and Gao, G. (2014). Empty Virions In AAV8 Vector Preparations Reduce Transduction Efficiency And May Cause Total Viral Particle Dose-Limiting Side-Effects. Mol. Ther. Methods Clin. Dev. *1*, 20139.
16. Mingozzi, F., Anguela, X.M., Pavani, G., Chen, Y., Davidson, R.J., Hui, D.J., Yazicioglu, M., Elkouby, L., Hinderer, C.J., Faella, A., et al. (2013). Overcoming pre-existing humoral immunity to AAV using capsid decoys. Sci. Transl. Med. *5*, 194ra92.
17. Manno, C.S., Pierce, G.F., Arruda, V.R., Glader, B., Ragni, M., Rasko, J.J., Ozelo, M.C., Hoots, K., Blatt, P., Konkle, B., et al. (2006). Successful transduction of liver in hemophilia by AAV-Factor IX and limitations imposed by the host immune response. Nat. Med. *12*, 342–347.
18. Wu, T.L., Li, H., Faust, S.M., Chi, E., Zhou, S., Wright, F., High, K.A., and Ertl, H.C. (2014). CD8+ T cell recognition of epitopes within the capsid of adeno-associated virus 8-based gene transfer vectors depends on vectors' genome. Mol. Ther. *22*, 42–51.
19. Cao, W., Dong, B., Firrman, J., Moore, A.R., Wang, Q., Roberts, S.A., and Weidong, X. (2014). 460. A novel factor FVIII variant enhanced secretion for gene therapy of hemophilia A. Mol. Ther. *22*, S176.
20. Lock, M., Alvira, M.R., and Wilson, J.M. (2012). Analysis of particle content of recombinant adeno-associated virus serotype 8 vectors by ion-exchange chromatography. Hum. Gene Ther. Methods *23*, 56–64.

21. Zelenaia, O.M.F., Hauck, B., Zhou, S., High, K.A., and Wright, J.F. (2011). A Capsid Composition Variant Containing four VP Proteins Caused by a Novel Start Codon at Position 219 of the Nucleotide Sequence of AAV8 Cap. Mol. Ther. *19*, 403.

22. Kapranov, P., Chen, L., Dederich, D., Dong, B., He, J., Steinmann, K.E., Moore, A.R., Thompson, J.F., Milos, P.M., and Xiao, W. (2012). Native molecular state of adeno-associated viral vectors revealed by single-molecule sequencing. Hum. Gene Ther. *23*, 46–55.

23. Martino, A.T., Basner-Tschakarjan, E., Markusic, D.M., Finn, J.D., Hinderer, C., Zhou, S., Ostrov, D.A., Srivastava, A., Ertl, H.C., Terhorst, C., et al. (2013). Engineered AAV vector minimizes in vivo targeting of transduced hepatocytes by capsid-specific CD8+ T cells. Blood *121*, 2224–2233.

24. Allay, J.A., Sleep, S., Long, S., Tillman, D.M., Clark, R., Carney, G., Fagone, P., McIntosh, J.H., Nienhuis, A.W., Davidoff, A.M., et al. (2011). Good manufacturing practice production of self-complementary serotype 8 adeno-associated viral vector for a hemophilia B clinical trial. Hum. Gene Ther. *22*, 595–604.

25. Chen, L., Lu, H., Wang, J., Sarkar, R., Yang, X., Wang, H., High, K.A., and Xiao, W. (2009). Enhanced factor VIII heavy chain for gene therapy of hemophilia A. Mol. Ther. *17*, 417–424.

26. Dong, B., Nakai, H., and Xiao, W. (2010). Characterization of genome integrity for oversized recombinant AAV vector. Mol. Ther. *18*, 87–92.

27. Dong, B., Moore, A.R., Dai, J., Roberts, S., Chu, K., Kapranov, P., Moss, B., and Xiao, W. (2013). A concept of eliminating nonhomologous recombination for scalable and safe AAV vector generation for human gene therapy. Nucleic Acids Res. *41*, 6609–6617.

28. Wang, Q., Dong, B., Firrman, J., Roberts, S., Moore, A.R., Cao, W., Diao, Y., Kapranov, P., Xu, R., and Xiao, W. (2014). Efficient production of dual recombinant adeno-associated viral vectors for factor VIII delivery. Hum. Gene Ther. Methods *25*, 261–268.

OMTM, Volume *4*

Supplemental Information

Syngeneic AAV Pseudo-particles Potentiate Gene Transduction of AAV Vectors

Qizhao Wang, Biao Dong, Katie A. Pokiniewski, Jenni Firrman, Zhongren Wu, Mario P.S. Chin, Xiongwen Chen, LinShu Liu, Ruian Xu, Yong Diao, and Weidong Xiao

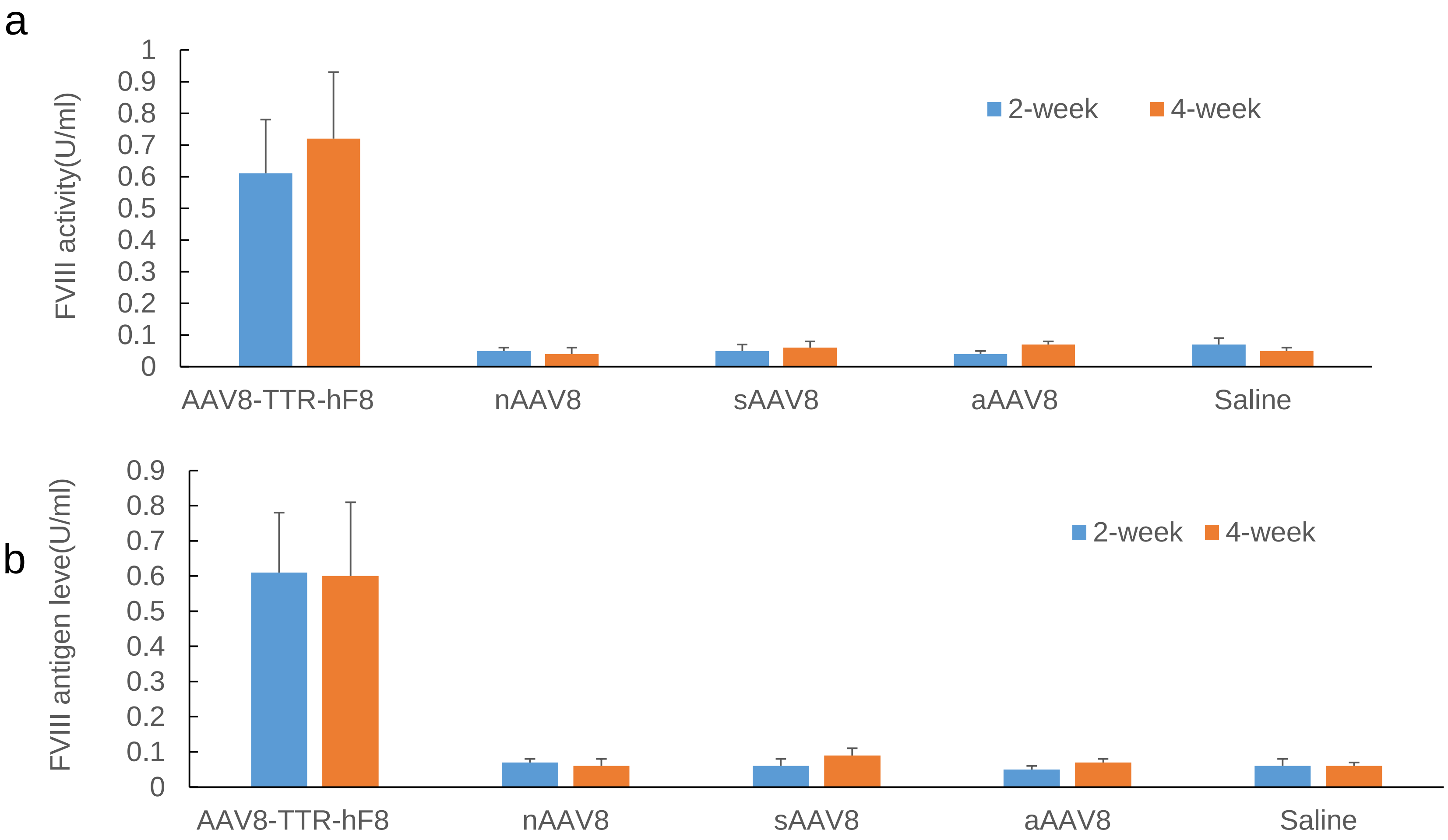

Figure S1. sAAV8 alone failed to express transgene *in vivo*. Blab/C mice were i.v. injected with AAV8-TTR-hF8 vectors (2×10^{11} vg/mouse) or 9X of AAV8 pseudo-vectors (nAAV8, sAAV8 and aAAV8). Transgene expression was detected by aPTT (a) and Elisa (b) at different points. nAAV, null AAV pseudo-vector; sAAV, syngeneic AAV pseudo-vector; aAAV, allogeneic AAV pseudo-vector.

Table S1. Probes for hybridization of AAV-TTR-hF8 and AAV-hHC genomes

Probe name	Targeting elements	Probe sequences	Location at AAV-hHC	Location at AAV-TTR-hF8
A	ITR	cgagcgagcgcgcagagagggagtggccaa-DIG	96-125; 3643-3672	96-125; 5051-5080
B	ApoE	acgcgtctgcaggctcagaggcacacagga-DIG	146-175	/
C	hHC	tccttgatgcaggatagggatgctgcatct-DIG	1593-1622	1201-1230
D	PolyA	gagatctgtgtgttggttttttgtgtgcgg-DIG	3494-3523	4902-4931
E	TTR	tgtctgtctgcacatttcgtagagcgagtg-DIG	/	151-180
F	hLC	agaaactgcagggctccctgcaatatccag-DIG		3565-3494

Table S2. qPCR primers for titration of AAV vectors

Primer pairs	Targeted genome	Forward primer	Reverse primer	PCR product (bp)	Location at AAV-hHC	Location at AAV-TTR-hF8
1	AAV-hHC; AAV-TTR-hF8	CGGCCTCAGTGAGCGA	GGAACCCCTAGTGATGGAGTT	62	83-144	83-144
2	AAV-hHC	AGTGGCCAACTCCATCACTA	GAGCCCAGAAACTCCTGTGT	71	117-188	
3	AAV-hHC	GCCTCTGAAGTCCACACTGA	AGGGCTGTGTGTTTGCTGT	92	243-344	
4	AAV-hHC	ACCTCCAACATCCACTCGAC	GCGTCGACCACACTACCTAA	86	393-478	
5	AAV-hHC; AAV-TTR-hF8	TCCACCTGCTTCTTTCTGTG	TCCACTGCACCCAGGTAGTA	74	909-982	517-590
6	AAV-hHC; AAV-TTR-hF8	CTGAAATGGATGTGGTCAGG	AGTCCCAGTCCTCCTCTTCA	126	2008-2133	1616-1741
7	AAV-hHC; AAV-TTR-hF8	AGTTCCAAGCCTCCAACATC	GGAAGTCAGTCTGTGCTCCA	126	2770-2895	2378-2503
8	AAV-hHC	CAGAATCCACCAGTCTTGA	GTCAATTTCCTCTTGATCTGAC	78	3180-3257	
9	AAV-hHC; AAV-TTR-hF8	CCTCTACTGACTCGAGAATAAA	GCCAACTCCATCACTAGG	95	3458-3552	4866-4690
10	AAV-TTR-hF8	AGTGGCCAACTCCATCACTA	ACCTTGCCTAGGGAGATTAG	94		117-210
11	AAV-TTR-hF8	CAGAATCAGCAGGTTTGGAG	GAACCACACACGGCACTTAC	144		261-404
12	AAV-TTR-hF8	CCAGATGGAAGATCCCACTT	GCTGAGCAGATACCATCGAA	124		3591-3714
13	AAV-TTR-hF8	GGCCATCAGTGGACTCTCTT	AATTCGAAGGTAGCGAGTCAG	123		4678-4800
qEGFP	AAV-CB-EGFP	TGACCCTGAAGTTCATCTGC	GAAGTCGTGCTGCTTCATGT	125	/	/
qCluc	AAV-CB-Cluc	TAAGACTTGCGGTATTTGCG	GCAGAGTCGTTCAGCTTCAG	135	/	/
